# Experience of clinical screening for COVID-19 among patients undergoing elective orthopedic surgeries: an alternative proposal

**DOI:** 10.1186/s13018-021-02217-8

**Published:** 2021-02-02

**Authors:** Edwarth Soler, Sammy Nicolás Farah, Valeria P. Bustos, Sofía Elizabeth Muñoz Medina, Jairo Fernando Gómez, Ernesto Martinez Lema, Carlos Álvarez Moreno

**Affiliations:** 1 Department of Orthopedics, Keralty Group, Clínica Universitaria Colombia, Bogotá, Colombia; 2 Department of Orthopedics, Keralty Group, Fundación Universitaria Sanitas, Bogotá, Colombia; 3 Faculty of Medicine, Pontificia Universidad Javeriana, Bogotá, Colombia; 4 Department of Epidemiology, Keralty Group, Fundación Universitaria Sanitas, Bogotá, Colombia; 5 Department of Orthopedics, Clínica Reina Sofia, Keralty Group, Bogotá, Colombia; 6 Department of Infectious Diseases, Sanitas-Keralty Group, Bogotá, Colombia

**Keywords:** Coronavirus, Orthopedic procedures, Outpatient surgical procedures

## Abstract

**Background:**

The coronavirus disease 2019 (COVID-19) pandemic is the largest global event in recent times, with millions of infected people and hundreds of thousands of deaths worldwide. Colombia has also been affected by the pandemic, including by the cancellation of medically necessary surgical procedures that were categorized as nonessential. The objective of this study was to show the results of the program implemented in two institutions in Bogotá, Colombia, in April 2020 to support the performance of elective essential and nonessential low- and medium-complexity orthopedic surgeries during the mitigation phase of the COVID-19 pandemic, which involved a presurgical clinical protocol without serological or molecular testing.

**Methods:**

This was a multicenter, observational, retrospective, descriptive study of a cohort of patients who underwent elective orthopedic surgery at two institutions in the city of Bogota, Colombia, in April 2020. We implemented a preoperative clinical protocol that did not involve serological or molecular tests; the protocol consisted of a physical examination, a survey of symptoms and contact with confirmed or suspected cases, and presurgical isolation. We recorded the types of surgeries, the patients’ scores on the medically necessary, time-sensitive (MeNTs) scale, the presence of signs, symptoms, and mortality associated with COVID-19 developed after the operation.

**Results:**

A total of 179 patients underwent orthopedic surgery. The average age was 47 years (Shapiro-Wilk, *P* = 0.021), and the range was between 18 and 81 years. There was a female predominance (61.5%). With regard to the types of surgeries, 86 (48%) were knee operations, 42 (23.5%) were hand surgeries, 34 (19%) were shoulder surgeries, and 17 (9.5%) were foot and ankle surgeries. The average MeNTs score was 44.6 points. During the 2 weeks after surgery, four patients were suspected of having COVID-19 because they developed at least two symptoms associated with the disease. The incidence of COVID-19 in the postoperative period was 2.3%. Two (1.1%) of these four patients visited an emergency department where RT-PCR tests were performed, and they tested negative for severe acute respiratory syndrome coronavirus 2 (SARS-CoV-2). No patients died or were hospitalized for symptoms of COVID-19.

**Conclusion:**

Through the implementation of a presurgical clinical protocol consisting of a physical examination; a clinical survey inquiring about signs, symptoms, and epidemiological contact with suspected or confirmed cases; and presurgical isolation but not involving the performance of molecular or serological diagnostic tests, positive results were obtained with regard to the performance of low- and medium-complexity elective orthopedic surgeries in an early stage of the COVID-19 pandemic.

**Level of evidence:**

IV.

## Background

The coronavirus disease 2019 (COVID-19) pandemic is the most critical global event that has occurred in recent times, affecting almost 20 million people and causing more than 700,000 deaths across five continents at the time of writing [1]. Healthcare systems have had to adapt inpatient and surgical services to meet the guidelines for providing care during the pandemic. As in other countries, the healthcare system in Colombia has had to redirect resources to increase the intensive care unit (ICU) capacity and the numbers of ventilators and health care personnel. The risk of contracting COVID-19, both for patients and healthcare personnel, has led to the cancellation of nonessential surgical procedures [2]. A study conducted in May 2020 by the COVID-19 Collaborative suggested that, globally, the specialty that would be forced to cancel the most procedures during the pandemic would be orthopedics. Colombia is one of the top five countries with the most postponed surgeries [3], leading to millions of dollars in losses in the healthcare sector, an increase in patient complications, a deterioration in patient quality of life, and an increase in their absence from work [2, 4].

Few studies have been conducted that provide evidence supporting either the postponement or performance of elective surgical procedures during the COVID-19 pandemic [4–7].

Our preoperative protocol for the prevention of the spread of COVID-19 includes preoperative isolation and a survey collecting information about the signs and symptoms of COVID-19 and the patient’s epidemiological contact history. Preoperative serological or molecular testing is not performed [8]. The decision not to perform testing was made with consideration of the degree of uncertainty that exists regarding the pandemic, the controlled environment in the operating rooms, and the documented limitations of the currently available diagnostic tests [9]. In Colombia, which is a country with limited resources, it was considered important to continue to perform elective low- and medium-complexity orthopedic surgeries.

With regard to the epidemiological context at the time the study was conducted (April 2020), Colombia was in the early mitigation phase, with an estimated 10% of the cases due to community transmission [10]. At the time, the prevalence of COVID-19 in Colombia was low, with fewer than 50 cases per 100,000 population, although strict lockdown measures were already being implemented throughout the country [10, 11]. By April 30, 6507 cases and 293 deaths had been reported, and Bogotá was the epicenter of the pandemic [12].

The objective of this study was to describe the results of the presurgical clinical protocol that was established in April 2020 in two institutions in Bogotá, Colombia, to allow the performance of elective low- and medium-complexity orthopedic surgeries during the mitigation phase of the COVID-19 pandemic; postoperative symptomatic cases, hospitalizations, ICU admissions, and mortality were analyzed.

## Methods

The present study was a multicenter, observational, descriptive, retrospective study of a cohort of patients undergoing elective orthopedic surgery in a high-complexity university clinic and an affiliated medium-complexity ambulatory elective surgery center (Clínica Universitaria Colombia and Centro Médico Puente Aranda) in the Keralty group, in Bogotá, Colombia, between April 1 and 30, 2020. Clínica Universitaria Colombia is a general reference center where COVID-19 and non-COVID-19 patients are treated and undergo operations. Puente Aranda ambulatory surgery center is a COVID-19 free institution.

All patients who were admitted for elective surgery were screened with a presurgical clinical protocol immediately prior to the procedure that included a complete physical examination, a survey of COVID-19-associated symptoms (fever, dyspnea, cough, muscle pain, dysgeusia, dysosmia, or diarrhea), and an assessment of any epidemiological history of contact with individuals with suspected or confirmed cases of COVID-19. The patients who were scheduled to undergo operations had been put into mandatory quarantine on March 20, 2020, which was when the city of Bogotá implemented a strict confinement policy [8, 13]. If any positive findings were identified when following this protocol, the procedure was cancelled, and the patient was referred to the emergency department for appropriate management. No patient was tested (antibodies or RT-PCR) for the presence of SARS-CoV-2 as part of our clinical protocol due to the limited availability of these tests in the country at the time of this study [8, 13].

Patients were not followed for more than 2 weeks after the surgery because the aim of the study was to determine the incidence of COVID-19 in this population in the perioperative period after the implementation of the presurgical clinical protocol. Based on the natural history of the disease and considering the maximum incubation period (14 days) [14], the development of signs and symptoms after this period would imply that the patient had contracted the disease after the operation. This study was not intended to investigate the rates of postoperative complications other than the development of COVID-19 during follow-up.

**Fig. 1:**
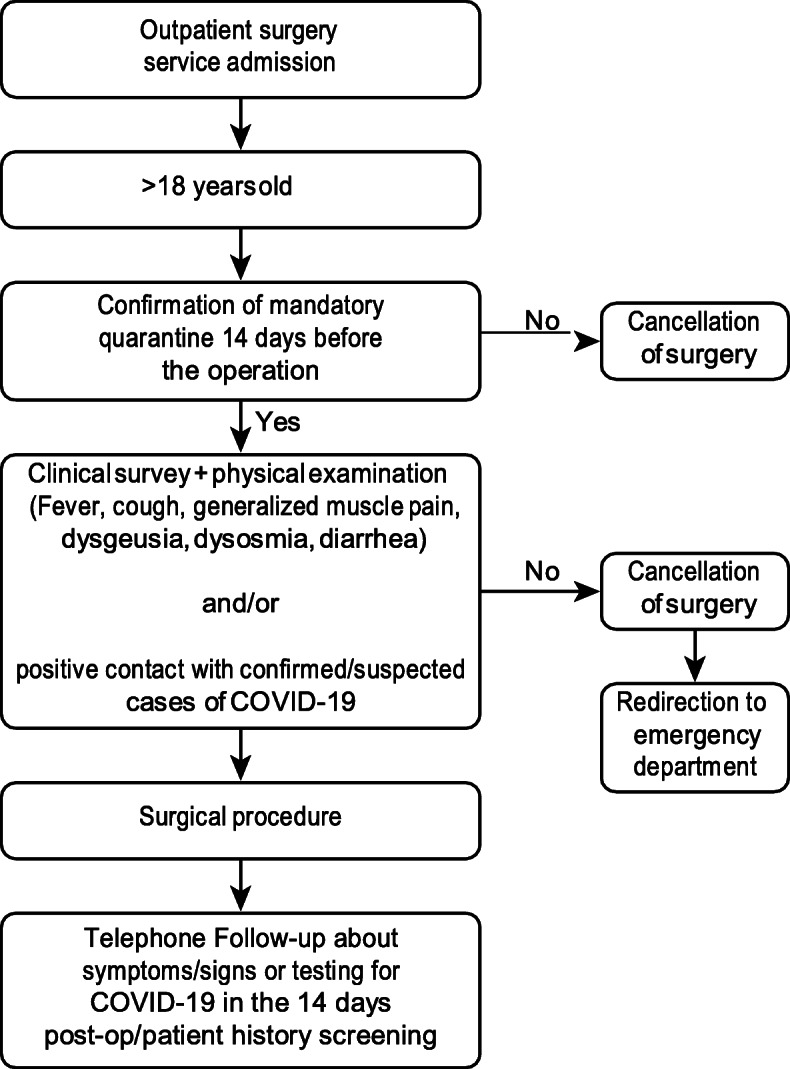
Study design flowchart

The included patients were those over 18 years of age who were candidates for elective orthopedic surgery in the two institutions mentioned above between April 1 and 30, 2020. Patients who were hospitalized at the time of surgery and those who had any positive indications of having COVID-19 during the presurgical screening were excluded (see Fig. 1)

The primary outcome was the incidence of confirmed or suspected COVID-19, which was described as a discrete variable. Other important variables were the type of surgery and the medically necessary time sensitive (MeNTs) scale score, which were defined as nominal and continuous categorical variables, respectively [7].

The following data were extracted: the demographic characteristics of the patients (age, sex, comorbidities, body mass index [BMI]), the presence of symptoms suggestive of COVID-19 in the 2 weeks following surgery, whether molecular or serological tests for SARS-CoV-2 were performed in the postsurgical period, the type of surgery performed, the anatomical location of the surgery, and the American Society of Anesthesiologist (ASA) class. The MeNTs scale [7] score was calculated retrospectively for each patient.

Patient data were collected from patient records and during routine postdischarge calls made by Keralty staff; the questions asked during the calls were modified due to the pandemic and asked about signs and symptoms of COVID-19 and the patient’s epidemiological history of contact with individuals with confirmed or suspected cases of COVID-19. Patients were also asked whether any diagnostic tests for SARS-CoV-2 had been performed in the 14-day postoperative period and the results. In addition, patients who had undergone RT-PCR testing for SARS-CoV-2 were identified through the medical insurance (Sanitas) database.

A descriptive statistical analysis was conducted. Continuous variables were analyzed with central tendency and dispersion measures based on the normality of their distributions. Kurtosis and skewness values were used to evaluate the distribution of the data, and a normal distribution was indicated by values of 0 and 3, respectively. The normality of the distribution of the data was corroborated with the Shapiro-Wilk test. Since some variables were normally distributed and others were not, means and standard deviations are presented, and medians and interquartile ranges are reported for each continuous variable. Categorical variables are presented as frequencies and percentages. The mean MeNTs scale score and the standard deviation were calculated for each group of surgeries. Data analysis was performed using the SPSS (IBM—International Business Machine Corporation) version 25 statistical program.

## Results

In total, 179 adult patients who underwent elective orthopedic surgery at Clínica Universitaria Colombia (21%) and Puente Aranda Ambulatory Surgery Center (79%) during April 2020 were included in the study. The mean age was 47 years, with a standard deviation of 14.83 and a range from 18 to 81 years; a majority of the patients were female (61.5%) (see Table 1).

**Table 1:** Demographic variables

Variable	Number of patients	Data presentation
Age (years + SD) ^d^ (years, IQR)	179	47.5 ± 14.83 ^a^ 49 (36–57) ^b^
Sex ^c^		
Female	110	61.4%
BMI ^e^	179	26.5 ± 3.79 ^a^ 26 (IQR 24–29) ^b^
ASA class ^c^		
I	72	40.78%
II	101	56.42%
III	5	2.79%
IV y V	0	0%
Comorbidities		
Arterial hypertension ^c^	35	19.55%
Coronary disease ^c^	3	1.68%
Diabetes ^c^	11	6.15%
Hypothyroidism ^c^	16	8.94%
Cancer ^c^	2	1.12%
Pulmonary disease ^c^	5	2.80%
CKD^c^	0	0%
Rheumatoid arthritis ^c^	3	1.69%
Others ^c^	2	1.12%
HIV ^c^	0	0.%
Smoking ^c^	29	16.20%
Surgery location ^c^		
Puente Aranda	142	79.33%
Clínica Colombia	37	20.67%
Operation duration (min)^f^	179	69.4 ± 39.2 ^a^ 60 (IQR 50-80) ^b^
Postoperative hospitalization ^c^		
No	175	97.77%
Yes	4	2.23%
Anesthesia type ^c^		
General	71	39.66%
Spinal	72	40.22%
Blockade	5	2.79%
Local	4	2.23%
Combined	27	15.08%
Contact with individuals with COVID-19 ^c^	5	2.79%
Presence of symptoms ^c^	4	2.23%
Medical attention ^c^	2	1.12%
Developed COVID-19^c^	0	0%
Tests for COVID-19 ^c^	2	1.12%

Note: Since there are some variables with normal distributions and others with nonnormal distributions, means and standard deviations and medians and interquartile ranges are reported as the measures of central tendency and dispersion

*BMI* body mass index, *COVID-19* coronavirus disease 2019

^a^Values presented as the means and standard deviations

^b^Values presented as the medians and interquartile ranges

^c^Values presented as the frequencies and percentages

^d^Skewness = − 0.05, kurtosis = 2.40 and Shapiro-Wilk (Swilk) = 0.02 = nonnormal distribution

^e^Skewness = 0.28, kurtosis = 3.14 and Shapiro-Wilk (Swilk) = 0.20 = normal distribution

^f^Skewness = − 1.96, kurtosis = 5.14 and Shapiro-Wilk (Swilk) = 0.00 = nonnormal distribution

With regard to the clinical characteristics of the patients, 60% were overweight, with a median BMI of 26 kg/m^2^ (Swilk = 0.2), 19.5% had high blood pressure, and 16% had a history of smoking. In addition, 9% had hypothyroidism, 6% were diabetic, 4% had lung disease, 1.7% had coronary heart disease, 1.7% had collagen disease, and 1.7% had a history of cancer.

In the postoperative period, four patients were suspected of having COVID-19 because they developed at least two symptoms associated with the disease; the incidence of suspected cases of COVID-19 was 2.3%. Two (1.1%) of these four patients consulted an emergency department where RT-PCR tests were performed, which were negative for SARS-CoV-2. No patients died or were hospitalized for symptoms associated with COVID-19.

Of the procedures performed, 86 (48%) were knee surgeries, 42 (23.5%) were hand surgeries, 34 (19%) were shoulder surgeries, and 17 (9.5%) were foot and ankle surgeries (see Table 2). No patients underwent hip or spine surgeries. The institutions did not allow hip and spine surgeries to be performed during the COVID-19 pandemic because they involve longer operation durations and are associated with higher risks of bleeding, morbidity, and ICU admission in the postoperative period.

**Table 2:** MeNTs scale score stratified by the type of surgery and anatomical surgical location

Type and location of surgery	Frequency (percentage)	Average MeNTs scale score
Shoulder		
Rotator cuff	29 (16.2)	47.1
Instability	2 (1.1)	44.7
Arthroplasty	1 (0.6)	59.0
Others	2 (1.1)	46.0
Subtotal	**34 (19.0)**	**47.2** ± **2.7** ^a^
Hand		
Carpal tunnel	10 (5.6)	42.3
Trigger finger	1 (0.6)	41.0
Arthrodesis	2 (1.1)	44.5
Tenosynovectomy	1 (0.6)	48.0
Ganglion cyst	7 (3.9)	41.1
Fractures	10 (6.1)	35.4
Removal of osteosynthesis material	4 (2.2)	44.0
Others	7 (3.9)	43.0
Subtotal	**42 (23.5)**	**40.3** ± **4.9** ^a^
Knee		
Arthroplasty	15 (8.4)	49.0
Anterior cruciate ligament	22 (12.3)	44.4
Meniscoplasty	25 (14.0)	46.4
Patellar malalignment	17 (9.5)	46.7
Removal of osteosynthesis material	1 (0.6)	44.0
Others	6 (3.4)	45.3
Subtotal	**86 (48.0)**	**46.4** ± **2.4** ^a^
Foot/ankle		
Forefoot	5 (2.8)	48.4
Midfoot	1 (0.6)	45.0
Hindfoot	2 (1.1)	44.0
Foot fractures	4 (2.2)	32.5
Onychectomy	2 (1.1)	32.5
Removal of osteosynthesis material	3 (1.7)	40.3
Subtotal	**17 (9.5)**	**41.8** ± **6.5** ^**a**^
Total	**179 (100)**	**44.7** ± **4.6** ^a^

*MeNTs* medically necessary time-sensitive

^a^Values presented as the means and standard deviations

The mean score for all patients on the MeNTs scale was 44.6 (SD ± 4.6) points. Only one patient surpassed the established safety limit of 55 points, scoring 59 points. The results stratified by anatomical location and procedure are shown in Table 2. Concerning the type of anesthesia, 70 patients (40%) underwent general anesthesia, and the remaining patients received spinal anesthesia, blockades, or mixed anesthesia. With regard to the ASA class, 73 (40.8%) patients were classified as ASA I, 101 patients (56.4%) were classified as ASA II, and five patients (2.8%) were classified as ASA III; no patients were classified as ASA IV or V. Some details of 15 patients who underwent knee arthroplasties, which are considered theoretically complex knee surgeries, should be noted. These patients had an average age of 68.9 (SD, 3.6) years with a range between 56 and 79 years, and an average BMI of 27.4 kg/m^2^ (SD ± 3.6). In this subgroup of patients, the distribution of ASA classes was as follow: I (20%), II (67%), and III (13%). The mean MeNTs score was 49.6 (SD 1.45).

## Discussion

The present study reports the experience of performing elective orthopedic surgeries, both essential and nonessential, of low and medium complexity after implementing a presurgical protocol that involved isolation and an epidemiological survey but not serological or molecular testing in an early stage of the COVID-19 pandemic.

The results obtained were satisfactory in that none of the patients developed serious symptoms, needed to be hospitalized, or died. This study showed that when laboratory tests are unavailable for various reasons, a clinical protocol can be an alternative means of screening patients before performing elective surgeries of low and medium complexity.

This study gave similar results to another study conducted by Ruggieri et al. in which they performed extensive swabs for SARS-CoV-2 and found only 2 positive cases out of the more than 200 orthopedic surgical procedures performed [15].

Initial studies in China showed poor results in patients who underwent surgery during the incubation period of the disease, with a mortality rate of 21%. On the other hand, a more recent study conducted in Spain about gastrointestinal surgery showed, in its elective surgery arm, only one patient developed COVID-19 out of the 97 patients who underwent operations; this patient recovered successfully. These findings were in accord with our results [4], although gastrointestinal surgery patients could be at a higher risk than orthopedic patients for COVID-19, and that study was conducted during the peak of the pandemic in Spain.

A paper by Gruskay et al. [16] suggested the need for widespread diagnostic testing of all patients admitted for essential surgery due to the high frequency of asymptomatic cases; however, that study did not perform laboratory testing for patients undergoing outpatient surgery. Another study carried out in Italy, in an orthopedic reference center, suggested little compromise in terms of quality and efficiency of medical care to these patients using a protocol similar to the one implemented in this study, with the difference of laboratory testing and computed chest tomography being used [17].

The patients included in this study were retrospectively evaluated with the MeNTs scale. This scale was calculated for each patient based on the pathology, the corresponding procedure, and clinical characteristics, yielding an mean score of 44.7 (SD 4.6) points, which was reasonable with regard to the safety cutoff value of 55 points suggested by the authors of another study [7]. The findings of our study suggest the importance of the use of this scale during the COVID-19 pandemic and support its effectiveness in the field of orthopedic surgery.

The present study was conducted during the early stages of the pandemic in Colombia; however, given the complex dynamics of epidemics, it is likely that similar circumstances will be observed again over the course of the pandemic [18]. Based on the fact that the pandemic is occurring in waves and the possibility of it spreading to new geographic regions as well as the potential for future pandemics, it is important to consider effective protocols.

The main justification for continuing to perform elective orthopedic surgeries is that although they are classified as elective surgeries, they are actually medically necessary surgeries that allow patients to have a better quality of life, decrease or eliminate pain and disabilities, and reduce societal costs by reducing absences from work [7, 19]. The increased cost imposed on the health system by the increase in complications associated with the delay in the surgical management of pathologies is also a reason to continue performing these procedures. Another important reason for continuing elective orthopedic surgeries is that the unclear duration of the pandemic, coupled with the absence of a vaccine and effective treatment, makes the indefinite postponement of surgeries untenable.

Yet another key reason to continue with elective surgery is resident education, studies suggest that while most challenges to resident’s education brought on by the pandemic can be overcome, there are certain elements of the intra-operative experience that are irreplaceable [20].

Among the limitations of this study is that it only provides insight into the early stage of the pandemic. Furthermore, the protocol had limited ability to detect asymptomatic COVID-19 patients and those in the incubation period. Another limitation is the fact that no PCR testing was performed to confirm the performance of the protocol. The results suggest that it was safe to perform elective orthopedic surgeries of low and medium complexity in the early stages of the pandemic, but because of the nature of this study, these results cannot be generalized to other populations. The findings also suggest the effectiveness of the presurgical protocol with regard to preventing patients with clinical suspected cases of COVID-19 from undergoing surgical procedures, which could increase the risk of postsurgical complications [21].

Future studies in later stages of the pandemic and involving other levels of surgical complexity are needed to more accurately establish the safety of elective orthopedic procedures.

## Conclusions

The present study described our experience performing low- and medium-complexity elective orthopedic surgeries in an early stage of the COVID-19 pandemic. The selection of patients through a clinical protocol that included a physical examination, a survey collecting information about symptoms and the patient’s epidemiological history of contact with people with confirmed or suspected cases of COVID-19, and presurgical isolation but did not include serological or molecular testing, yielded good results with regard to the safety of performing elective surgery in this group of patients during the pandemic.

## Data Availability

The datasets used and analyzed during the current study are available from the corresponding author on reasonable request: edsp33@yahoo.com.

## Abbreviations

COVID-19: Coronavirus disease 2019
MeNTs: Medically necessary time-sensitive
Swilk: Shapiro-Wilk
RT-PCR: Reverse-transcription polymerase chain reaction.
SARS-CoV-2: Severe acute respiratory syndrome coronavirus 2
ICU: Intensive care unit
BMI: Body mass index
ASA: American Society of Anesthesiologists
IBM Corp.: International Business Machine Corporation
SD: Standard deviation
IQR: Interquartile range
CKD: Chronic kidney disease
HIV: Human immunodeficiency virus
